# Sensitivity and Bias in Face-Emotion Labeling: Replication and Extension to Youth With Irritability and Anxiety

**DOI:** 10.1016/j.jaacop.2025.09.005

**Published:** 2025-10-22

**Authors:** Katherine Y. Kim, Joel Stoddard, Sofia I. Cárdenas, Parmis Khosravi, Katharina Kircanski, Matt Jones, Daniel S. Pine, Melissa A. Brotman, Simone P. Haller

**Affiliations:** a National Institute of Mental Health, National Institutes of Health, Bethesda, Maryland; b University of Colorado, Anschutz Medical Campus, Aurora, Colorado; c University of Colorado Boulder, Boulder, Colorado

**Keywords:** face-emotion labeling, mood and anxiety disorders, computational modeling, children and adolescents, fMRI

## Abstract

**Objective:**

Pediatric anxiety and irritability are common, impairing, co-occurring symptoms. Biases in interpreting ambiguous face-emotions have been linked to both phenotypes. Here, we assessed whether biases represent a shared cognitive and neural profile. In addition, we attempted replication of prior age associations and group behavioral differences in face-emotion labeling.

**Method:**

In a cross-sectional functional magnetic resonance imaging study at a research facility at the National Institute of Mental Health, we used a variant of a drift diffusion model to decompose perceptual and cognitive components of binary happy–angry decisions to ambiguous face-emotions. A total of 95 participants (mean age = 14.11, SD = 3.07, range = 8-22, male = 50.9%) contributed to the analyses. In all, 65 participants did not complete the scan or meet data quality thresholds. Participants had a primary diagnosis of disruptive mood dysregulation disorder (DMDD; n = 27), attention-deficit/hyperactivity disorder (n = 23), oppositional defiant disorder (n = 2), anxiety disorder (n = 19), or no current diagnosis (n = 24), resulting in a wide range of anxiety and irritability symptom levels.

**Results:**

No significant associations between the computational modeling parameter indexing bias and dimensionally assessed anxiety or irritability emerged (*r*[84] < −0.15, *p* > .05). Parent- and child-rated irritability, but not anxiety, was associated with increased neural responses to overtly angry faces, most notably in the motor cortex (voxel-wise *p* < .005). We successfully replicated prior age-associated increases in sensitivity and differences in bias between youth with DMDD and controls.

**Conclusion:**

Increased neural engagement of the bilateral motor cortex likely reflects increased vigor in responding to overtly angry faces, possibly indexing approach behavior. Replication of prior findings increases confidence in the robustness of these associations.

## Plain language summary

Youth experiencing high levels of anger or anxiety are thought to interpret ambiguous facial expressions as hostile. In this study, 95 youth aged 8 to 22 years completed a task that assessed their interpretations of facial expressions while measuring brain activation. Youth with higher levels of anxiety or anger did not interpret faces as more hostile. However, when responding to angry faces, youth with higher levels of anger showed increased activation in areas of the brain involved in coordinating actions, which may translate to more aggressive responses in daily life.

Pediatric irritability and anxiety are common, impairing, and often co-occurring problems.^1^, ^2^, ^3^ Both phenotypes relate to poor educational and socioeconomic attainment as well as adult psychopathology.^4^, ^5^, ^6^, ^7^ In the community, individual differences in anxiety and irritability are correlated and distributed continuously.^8^, ^9^, ^10^, ^11^ Previous studies have provided evidence that youth with irritability and/or anxiety exhibit biases in the processing of social cues: they orient preferentially toward angry faces^12^^,^^13^ and interpret ambiguous facial expressions as hostile.^14^^,^^15^ However, largely because of siloed research approaches, it remains unclear whether these biases represent a shared cognitive profile or are driven by one symptom dimension over the other.

Irritability is defined as increased proneness to anger relative to that of peers^16^; in its severe form, it has been codified in the *DSM-5* as disruptive mood dysregulation disorder.^17^ Anxiety refers to excessive and persistent feelings of fear and worry. Although both irritability and anxiety are characterized by intense, negative-valence affective states,^18^^,^^19^ the behavioral outputs differ. Irritability involves approach behavior such as reactive aggression,^20^^,^^21^ whereas anxiety involves avoidance.^22^ Examining whether interpretation bias represents a shared cognitive pathway related to both phenotypes informs the search for treatment targets for behavioral interventions.

Mapping treatment targets requires not only precision in the clinical phenotype, but also precision in the cognitive and/or neural mechanism. In a prior publication,^23^ we applied a variant of a classic drift diffusion model (DDM)^24^ as an effective way to study differences in behavioral performance in face-emotion discrimination tasks that assess interpretation bias in healthy volunteers. This DDM variant parses perceptual and cognitive components of binary choice decisions to ambiguous face-emotions; specifically, it metricizes 2 processes: (1) the efficiency in perceptual sampling of subtle face-emotions, namely, sensitivity to changes in valence; and (2) the calibration of the evidence accumulation relative to what is assumed to be the evidence rate for the most ambiguous stimulus, namely, perceptual bias. In prior work,^23^ we demonstrated age-associated changes across ages 8 to 35 years in sensitivity but not perceptual bias. We also found more pronounced neural responses to more ambiguous face-emotion displays in the anterior insula with increased age, assessed cross-sectionally. Here, we aim to extend this work to examine associations between these metrics and levels of anxiety and irritability measured continuously in a transdiagnostic sample of youth.

Independent avenues of work have linked anxiety and irritability to perturbed amygdala, ventral visual stream, and lateral and medial prefrontal circuitry functioning during face-emotion labeling.^25^^,^^26^ Limited work in both domains has investigated neutral or ambiguous emotion displays; this work has identified spatially overlapping dysfunctions between the 2 phenotypes that may index biases in the subjective interpretation of ambiguous face-emotions.^27^, ^28^, ^29^ Inconsistencies across both prototypical and ambiguous face-emotion displays may be due to differences in task demands (eg, explicit or implicit emotion labeling), medication, patient or developmental status of participants, or measurement of clinical variables (diagnostic vs continuous approaches). Rarely have studies examined both phenotypes simultaneously. The 2 studies that have examined both domains of symptoms have shown aberrant activation patterns to be predominantly linked to irritability, not anxiety.^30^^,^^31^

### The Current Study

Biases in the interpretation of ambiguous social cues have been theorized as a key feature of emotional problems and externalizing behavior, with a causal role in symptom maintenance.^32^^,^^33^ In the current study, we first examined associations between individual differences in anxiety and irritability and computational metrics of sensitivity and bias in a transdiagnostic sample of youth and young adults. We expected both irritability and anxiety to associate with a bias toward angry interpretations to ambiguous face-emotions. Second, we investigated associations between individual differences and neural responses to face-emotion valence and ambiguity. These analyses were exploratory. We tentatively expected associations between irritability and activation patterns to ambiguous and angry face-emotions in perceptual (ventral visual stream), regulatory (lateral and medial prefrontal cortices), and motor regions. We hypothesized overlapping associations between anxiety and neural responses in perceptual and regulatory regions. Finally, we aimed to replicate age associations with sensitivity detailed in Haller *et al.*,^23^ as well as findings of differences in the interpretation of ambiguous face-emotions between youth with disruptive mood dysregulation disorder (DMDD0 and heathy control youth presented by Stoddard *et al.*^15^

## Method

### Participants

Participants were recruited via direct mailings, advertisements in local news outlets and community clinics, and community talks. An initial on-site evaluation visit determined eligibility for 2 different research characterization and treatment protocols recruiting healthy volunteers and several clinical groups based on a primary symptomatic target. Recruitment reflected the desire for a transdiagnostic sample with varying levels of irritability and anxiety, and included youth with a primary diagnosis of DMDD, attention-deficit/hyperactivity disorder (ADHD), one or more anxiety disorders (generalized, social or separation anxiety disorder), and/or oppositional defiant disorder (ODD) confirmed via the Kiddie-Schedule for Affective Disorders and Schizophrenia for School-Age Children—Present and Lifetime version (KSADS-PL),^34^ carried out by a doctoral- or master’s-level clinician. Diagnoses were reviewed by a board-certified psychiatrist or psychologist. A total of 160 participants enrolled in the study. The final sample consisted of 95 youth (mean age = 14.24 years, SD = 3.17, range = 8.43-22.31, male = 49.3%); 71 participants with a primary diagnosis of DMDD, ADHD, anxiety disorder, or ODD (mean age = 14.38 years, SD = 3.02, range = 9.32-21.33); and 24 healthy control youth (mean age = 13.84 years, SD = 3.61, range = 8.43-22.31). Data from these healthy volunteers were used in a prior publication.^23^ Exclusion criteria across all patient groups were as follows: lifetime history of psychosis, conduct disorder, autism spectrum disorder, posttraumatic stress disorder, and a full-scale IQ of <70 assessed via the Wechsler Abbreviated Scale of Intelligence^35^ or clinician semi-structured assessment and educational record review. Cardinal bipolar symptoms or substance abuse within the last 3 months was also exclusionary. Sample characteristics for the full sample are detailed in Table 1 and Table S1, available online, and contain sample characteristics for the subsample that completed additional functional magnetic resonance imaging (fMRI) scanning. Additional information on participant assessment and attrition can be found in Supplement 1, available online. Written consent/assent from parents/children was obtained prior the initiation of any study procedures, and families received monetary compensation for their participation. The National Institute of Mental Health (NIMH) Institutional Review Board approved the study.

**Table 1:** Sample Characteristics (N = 95)

Characteristic	Value
Age, mean (SD)	14.24 (3.2)
IQ, mean (SD)	115.41 (12)
Participant’s household's gross income, n (%)	
$25,000-$39,999	1 (1.1%)
$40,000-$59,999	2 (2.1%)
$60,000-$89,999	10 (10.5%)
$90,000-$179,999	29 (30.5%)
Over $180,000	22 (23.2%)
Highest level of education, n (%)	
Graduate professional degree, masters or above	32 (33.7%)
Standard college graduation	21 (22.1%)
Partial college, 1 year or more	12 (12.6%)
High school graduation	1 (1.1%)
Junior high school, grades 7-9	4 (4.2%)
Less than 7 years of school	1 (1.1%)
**Sex**, n (%)	
Female	50 (52.6%)
Male	45 (47.4%)
Primary diagnosis, n (%)	
ADHD	23 (24.2%)
Anxiety	19 (20.0%)
DMDD	27 (28.4%)
Healthy	24 (25.3%)
ODD	2 (2.1%)
Race, n (%)	
American Indian or Alaskan Native	1 (1.1%)
Asian	1 (1.1%)
Black or African American	11 (11.6%)
Multiple races	8 (8.4%)
White	72 (75.8%)
Unknown	2 (2.1%)
Ethnicity, n (%)	
Unknown	2 (2.1%)
Not Hispanic or Latino	81 (85.3%)
Hispanic or Latino	12 (12.6%)
Medications, n (%)	
None	47 (49.5%)
Antidepressants	
SSRI	21 (22.1%)
SNRI	4 (4.2%)
SARI	1 (1.1%)
Mood stabilizers	
AED	8 (8.4%)
Lithium	1 (1.1%)
Anti-anxiety	
Azapirone	1 (1.1%)
Benzodiazepine	1 (1.1%)
SGA	7 (7.4%)
Stimulant	34 (35.8%)
Non-stimulant ADHD	5 (5.3%)

Note: A total of 20 participants did not have IQ scores; 31 were missing household gross income data; 24 were missing highest level of education; 1 was missing ethnicity; and 7 were missing information about medication use. Participant’s education and household’s gross income: if the participant was a child or college student, the highest level of education and the income of the parent were considered. If the participant was an adult, their own highest level of education and income were considered. ADHD = attention-deficit/hyperactivity disorder; AED = antiepileptic drug; DMDD = disruptive mood dysregulation disorder; ODD = oppositional defiant disorder; SARI = serotonin antagonist and reuptake inhibitor; SGA = second-generation antipsychotic; SNRI = serotonin-norepinephrine reuptake inhibitor; SSRI = selective serotonin reuptake inhibitor.

### Clinical Measures

#### Anxiety Symptoms

Youth anxiety symptoms were assessed using the Screen for Child Anxiety Related Emotional Disorders (SCARED) by both youth and parent over a 6-month period.^36^ The SCARED consists of 41 items that describe anxiety disorder symptoms across the 5 subscale scores (generalized anxiety, panic, social anxiety, separation anxiety, school anxiety), with a total score ranging from 0 to 82. The SCARED has good internal consistency across the total score (α = 0.90) as well as for the subscale scores (α = 0.78-0.87).^37^ The SCARED also shows moderate to good test–retest reliability for the total score (parent: 0.86, child: 0.62).^38^ Parent- and child-reported total scores, collected within 3 months of the research visit, were calculated as a measure of anxiety symptoms.

#### Irritability Symptoms

Youth irritability symptoms were assessed using the Affective Reactivity Index (ARI) reported by youth and parent.^39^ The ARI queries temper outbursts and irritable mood across a 6-month period using 6 items, resulting in a summed total score ranging from 0 to 12. The measure has excellent internal consistency (parent: α = 0.92-0.93, child: α = 0.86-0.89) and good test–retest reliability (parent: 0.85, child: 0.78).^39^^,^^40^ Parent- and child-reported total scores, collected within 3 months of the research visit, were calculated as a measure of irritability symptoms.

### fMRI Face-Emotion Labeling Task

The fMRI compatible face-emotion labeling task was used in prior work.^23^ Participants were required to make binary happy–angry decisions to rapidly presented face-emotions. Face-emotions ranged from prototypically happy to angry morphed linearly across 15 face-emotion morphs, with the middle morph exhibiting the most ambiguity first generated by Penton-Voak and colleagues.^41^ Face-emotions were displayed by a composite male face from the Karolinska Directed Emotional Faces database.^42^ Face-emotion morphs were presented at random for 150 milliseconds, followed by a white noise mask for 250 milliseconds, followed by a fixation screen. Participants were asked to use a button press to indicate whether they believed the face to be “happy” (left hand) or “angry” (right hand) as quickly and accurately as possible. Inter-trial intervals (ITIs) were jittered, presenting a fixation cross, with a minimum of 500 milliseconds and an ITI distribution following an exponential decay curve. The presentation of the face morphs and the jitter orders were optimized and pseudo-randomized using AFNI’s make_random_timing.py program. The task consisted of 90 fixation-only trials along with the presentation of each stimulus 30 times at random, resulting in a total of 540 trials completed over 4 runs. Each run began and ended with a 10-second fixation period, to result in about 421 seconds. A task schematic can be found in Figure S1, available online.

### Computational Model

We applied a variant of the drift diffusion model DDM,^43^^,^^44^ validated in prior work.^23^ The model parses 2 primary metrics of interest: sensitivity to changing facial affect and bias in perceiving facial ambiguity, derived from 2-choice binary happy–angry face-emotion labeling tasks. DDMs partition decision-making components using response choice and reaction time distributions across task trials. The total decision time is encapsulated in 3 main components: (1) the decision process, (2) decision criteria, and (3) non-decision processes such as early sensory encoding and later motor response execution that flank decision processes. The decision process is represented by the drift rate parameter “*v*,” which describes the average rate of dynamic and noisy evidence accumulation toward a decision boundary (ie, a happy or angry decision) for each of the 15 face morphs (stimulus “*s*1” to “*s*15”). The decision criteria are represented by the model parameter “*a*” and indexes the distance between happy–angry decision boundaries; non-decision components are represented by the parameter “*t*_0_.” An additional parameter, “*z,*” denotes the starting point of evidence accumulation (0 < *z* < *a*); “*z*” indexes biases that reside outside the perceptual domain and are present prior to stimulus onset (eg, a predominant trial type/choice response).

In the current variant of the DDM, drift rate “*v*” was assumed to vary linearly across the range of stimuli (“*s*1” to “*s*15”), as established across a range of binary choice perceptual discrimination tasks in prior work^45^:

$$v\left(s\right)=v_{\mathrm{int}}+s\;*\;v_{\text{slope}}$$

Two measures of interest can be derived through this extension: sensitivity, represented by *v*_slope_, with the steepness of the slope reflecting the sensitivity to changing facial affect; and decisional bias, represented by the point at which the drift rate, *v*(*s*), is zero, that is, the point of maximum ambiguity during decision processing:

$$s_{\text{indiff}}=-v_{\mathrm{int}}\;/\;v_{\text{slope}}$$

In summary, we estimated 3 parameters per participant in addition to sensitivity and perceptual bias: response bias (*z*_r_ *= z/a*), boundary criterion (*a*), and non-decision time (*t*_0_). Custom Matlab code used to apply this model is available on GitHub (https://github.com/NIMH-SDAN/Comp-modeling-of-face-emotion-processing). Quantile probability plots comparing empirical and predicted response choices and for reaction time distributions for happy and angry judgments per face-emotion morph are shown in Figure S2, available online.

### Imaging Data Acquisition and Preprocessing

Participants scanned on one of two 3T General Electric Signa Discovery MR750 scanners (GE Medical Systems) with 32-channel head coils. Functional data was acquired via T2∗-weighted echoplanar sequences measuring blood oxygen level–dependent (BOLD) signal across 183 volumes at a resolution of 2.5 × 2.5 × 3 mm with the following specifications: repetition time = 2.3 seconds, echo time = 30 milliseconds, field of view = 24 cm, and flip angle = 70°. Anatomical data via a structural 3D magnetization-prepared rapid gradient-echo MRI sequence (MPRAGE) was collected at a resolution of 1 mm^3^ with the following specifications: echo time/inversion time of minimum full echo time/425, field of view = 25.6 cm, flip angle = 7°. Anatomic scans were used for co-registration with the functional data.

### Analysis of Functional Neuroimages

AFNI version 24.3^46^ was used to analyze neuroimaging data. Pre-processing followed state-of-the-art recommendations including despiking, slice-time correction, alignment of all volumes to the volume with the smallest fraction of outliers, nonlinear registration to the Montreal Neurological Institute (MNI) template space, spatial smoothing to 6.5 mm full width at half maximum kernel ensuring similar smoothness across scanners with additional specifiers, masking by the anatomical images, and intensity scaling to a mean of 100. Volumes were censored if at least one of following 2 conditions was met: (1) the sum head displacement (Euclidean norm of the derivative of the translation and rotation parameters) between 2 volumes exceeded 1 mm, and (2) the volumes contained over 10% of voxels timeseries signal outliers. Participants were removed from further analyses if (1) average motion per volume after censoring was >0.25 mm and/or (2) >15% of volumes were censored for motion or outliers. This resulted in the exclusion of 19 participants. We report results using a “highlight, not hide” approach^47^; suprathreshold results are highlighted with black contours, whereas subthreshold locations are opaque but visible. An extension of the cluster summary table is provided in Table S3, available online, where additional clusters at a reduced threshold are listed.

First-level general linear models controlled for 6 head motion parameters and were applied with generalized least squares timeseries fit with restricted maximum likelihood estimation of the temporal autocorrelation structure (3dREMLfit). For each participant, 15 regressors of interest were devised, representing each of the 15 face morphs. An additional regressor coded response omissions. Custom code for preprocessing is available on GitHub https://github.com/NIMH-SDAN.

### Statistical Analysis

#### Behavioral data

Trials with reaction times of <150 milliseconds were excluded from further analyses. To be included in further analyses, participants had to meet a threshold of at least 80% correct on the 2 most extreme happy and angry morphs.

We examined associations between individual differences in child- and parent-rated anxiety and irritability and computational metrics, specifically sensitivity and perceptual bias, using Pearson correlations Bonferroni corrected for multiple comparisons. In addition, we examined group differences in these metrics between youth with a primary diagnosis of DMDD, an anxiety disorder, and ADHD and healthy controls using 1-way analysis of variance (ANOVA). Post hoc pairwise *t* test results were Bonferroni corrected for multiple comparisons. The nature of the current transdiagnostic sample is such that most youth meet criteria for more than one disorder, making diagnostic analyses challenging. However, prior work has primarily compared diagnostic groups; we therefore included diagnostic analyses for consistency and comparison with prior work. In addition, in Supplement 2, Table S2, and Figure S3, available online, we include analyses using reporter-averaged (ie, parent–child averaged clinical measures) analyses.

### Imaging Data

To investigate associations between clinical measures and neural responses to face-emotion valence and ambiguity, we applied 4 whole-brain linear mixed-effects models using 3dLMEr in AFNI.^48^ The 2 face morph dimensions of interest, happy–angry valence and ambiguity, were modeled via a linear and quadratic contrast centered on the middle morph; individual level estimates were weighted in 2 coefficients on the group level. In 4 models, we examined interactions between these 2 slope terms with either child- or parent-rated anxiety or irritability, with age, sex, scanner, and motion (fraction of censored data) as additional covariates. In Supplement 3, available online, we repeat the analyses using medication load as an additional covariate. All analyses were conducted across a whole-brain mask of voxels in which at least 90% of participants had signal. The voxel-wise *p* value threshold was .005, Bonferroni corrected to a family-wise error rate of α = 0.05/4 = 0.0125 using AFNI’s 3dClustSim (-acf, NN = 1, 2-sided), resulting in *k* = 93 voxels.

Post hoc illustrations depict associations between linear and quadratic coefficients per participant and the individual difference measure from extracted data of significant clusters. A median split of the individual difference measure was used for visual illustration of the results. Exploratory supplemental analyses (Supplement 4, available online) examined associations between neural coefficients for significant clusters and select computational parameters to investigate links between activation patterns and specific aspects of the decision-making process.

### Replication of Prior Work

Our setup allowed us to attempt replication of 2 prior behavioral findings in an independent sample: (1) age-associations in sensitivity detailed in Haller *et al.*,^23^ and (2) findings of differences between youth with DMDD and heathy control youth presented by Stoddard *et al.*^15^ We followed exact procedures detailed in prior work for each of these findings; details can be found in Supplement 5, available online.

## Results

### Associations Between Computational Parameters and Child- and Parent-Rated Anxiety and Irritability

Cross-informant correlations, that is, associations between child and parent ratings, were in the medium-to-large range for both measures: anxiety: *r*_p_(83) = 0.58, *p* < .001; irritability: *r*_p_(83) = 0.56, *p* < .001; cross-measure. Correlations between anxiety and irritability also showed a medium-to-large correlation for each informant: child: *r*_p_(84) = 0.46, *p* < .001, parent: *r*_p_(83) = 0.50, *p* < .001). There were no significant associations between the DDM-derived measures of sensitivity and perceptual bias with child- or parent-rated clinical measures (statistical values in Table 2) and no other significant associations between estimated parameters (*t*_0_*,z*_r_*,a*) and clinical measures emerged (all |*r*_ps_|< −0.20, *p* > .06).

**Table 2:** Associations Between Computational Metrics and Clinical Measures

	Anxiety–child, *r*_p_(85), *p*	Anxiety–parent, *r*_p_(84), *p*	Irritability–child, *r*_p_(91), *p*	Irritability–parent, *r*_p_(84), *p*
Sensitivity: *v(s)*	–0.079, .468	–0.036, .741	–0.121, .248	0.022, .840
Perceptual bias: *s*_*indiff*_	0.020, .855	0.007, .946	0.098, .352	–0.154, .158

Additional diagnostic analyses testing differences in perceptual bias using 1-way ANOVA found a significant main effect for diagnostic group (*F*_3,89_ = 3.799, *p* = .013). Post hoc multiple comparison corrected pairwise *t* tests showed a significant difference only between youth with a primary diagnosis of DMDD compared to healthy controls (mean for DMDD =7.52, SD for DMDD = 0.95; mean for controls = 8.48, SD for controls = 1.40, *p* = .024); youth with DMDD exhibited a perceptual bias relatively shifted toward angry judgements. A second ANOVA testing for significant differences in sensitivity found no main effect of primary diagnosis (*F*_3,89_ = 0.95, *p* = .42).

### Associations Between Neural Responses to Face-Emotion Valence and Ambiguity and Clinical Measures

**Figure 1:**
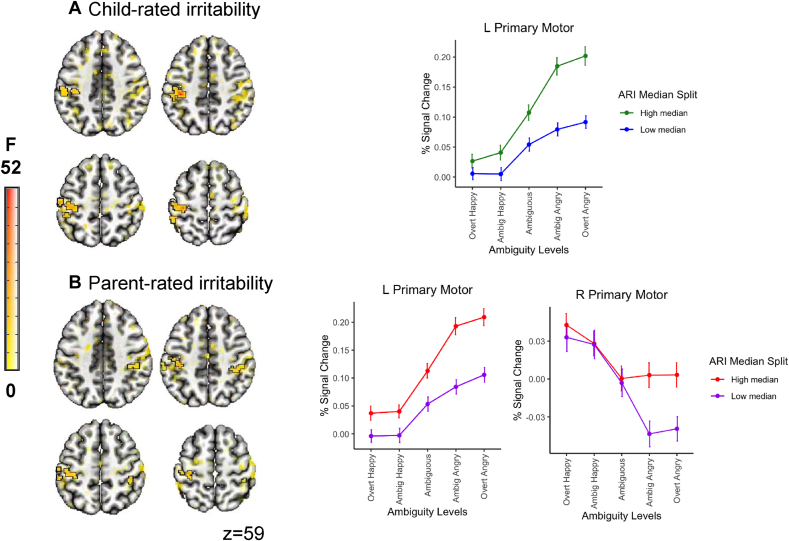
Associations Between Percent Signal Change to Valence (Modeled as a Linear Slope in the Voxel-wise Model) and Child- and Parent-Rated Irritability in a Transdiagnostic Sample of Youth ***Note:****For visualization, a median split was performed on the irritability variable. Signal change estimates are averaged across 3 adjacent face morphs for display purposes.*

Only the models examining associations between linear and quadratic activation patterns and irritability revealed significant interactions between the clinical variable and the within-subject slopes at adequate thresholding. Specifically, the left (for child-rated irritability) and the left and right (for parent-rated irritability) primary motor cortex showed an interaction between irritability and the linear morph term. Post hoc decomposition of these interactions revealed that neural activation to overt angry morphs increased with increasing levels of irritability (child: left primary motor: *F*_1,1060_ = 33.34, *p* < .001, *r*_p_= 0.355; parent: left primary motor: *F*_1,1060_ = 27.00, *p* < .001, *r*_p_ = 0.358, right primary motor: *F*_1,1060_ = 21.73, *r*_p_= 0.382, *p* < .001), as summarized in Table 3 and Figure 1).

**Table 3:** Summary of Clusters Showing a Significant Association Between the Linear Contrast Coding Stimuli Valence and the Parent- and Child-Rated Clinical Measures

ROI overlap and location	Cluster size		Coordinates (center of mass)			Coordinates (at peak)			Mean F	SEM	Max Int	Post hoc^a^
	*k*	mm^3^	CM LR	CM PA	CM IS	MI LR	MI PA	MI IS				
Primary clusters (*p* = .005)												
Irritability, child-report												
L postcentral gyrus (65.6%), L inferior parietal lobule (20.3%), L precentral gyrus (10.6%)	305	4765.625	–42.7	–28.1	53	–37.5	–29.2	48	15.141	0.4169	51.116	0.355
Irritability, parent-report												
L postcentral gyrus (72.2%), L precentral gyrus (9.3%), L inferior parietal lobule (7.4%)	124	1937.5	–44.8	–27.1	52.6	–35	–26.8	58	12.338	0.3244	25.149	0.358
R postcentral gyrus (58.4%), R supramarginal gyrus (21.1%), R inferior parietal lobule (12.2%)	97	1515.625	39.8	–33.2	47.2	40	–34.2	50.5	10.924	0.2565	18.442	0.382
Anxiety, child-report												
–												
Anxiety, parent-report												
–												

Note: Cluster-corrected voxel-wise linear mixed-effects model results are presented here, summarizing regions showing a significant linear trend-by-clinical measure interaction. Location lists regions in descending order based on proportion overlap with cluster. CM = center of mass of cluster; IS = inferior–superior (z); k = Number of voxels in cluster; LR = left–right (x); MI = max intensity (peak); mm^3^ = cubic millimeters (cluster volume); PA = posterior–anterior (y); ROI = region of interest.

a Post hoc values represent associations between the individual-level linear coefficient and the clinical measure.

### Replication of Prior Work

Prior work on associations between age and sensitivity examined a cross-sectional sample of healthy volunteers with an age range of 8 to 35 years; here, we had a smaller age range of 8 to 22 years. The healthy volunteers, who were part of the prior publication, were excluded for this independent replication; hence, the replication included only youth with a primary diagnosis of an emotional or behavioral disorder. The relationship between DDM-derived sensitivity and age in 76 youth was significant (*r*_p_[74] = 0.265, *p* = .02).

**Figure 2:**
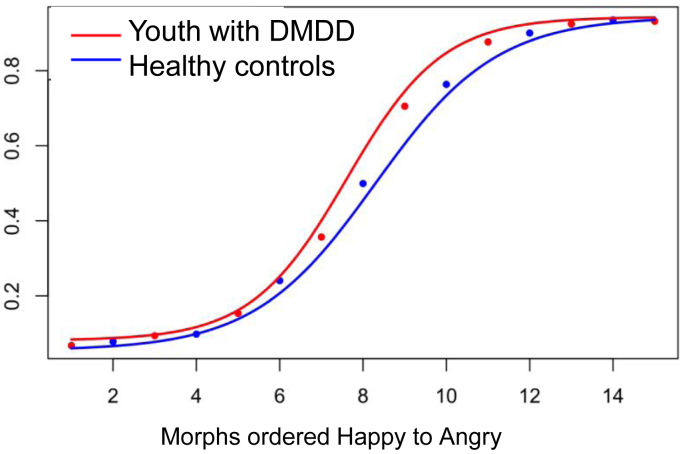
Mean Proportions of Angry Judgments Are Plotted Against Face-Emotion Morphs, Ordered From Happy to Angry ***Note:****For each group, solid line represents the fitted 4-parameter logistic curve. Relative to healthy volunteer youth, youth with disruptive mood dysregulation disorder (DMDD) have a bias toward judging ambiguous morphs as angry (*p *< .001).*

Stoddard *et al.*^15^ demonstrated a bias in interpreting ambiguous face-emotions in youth with DMDD compared to healthy control youth. Specifically, using identical procedures, namely, fitting a 4-parameter logistic regressor to the group level data, we replicated a significant difference in the inflection point of the logistic curve, adjusted for the maximum probability of either judgment (youth with DMDD: n = 27, healthy controls: n = 24). Youth with DMDD had a lower inflection point, indicating that they switched their judgment from predominantly happy to predominantly angry earlier along the face morph continuum (participants with DMDD: b[SE] = 7.65[0.06]; healthy volunteers: b[SE] = 8.33[0.08]; *t*(50) = −6.806, *p* < .001) (Figure 2).

## Discussion

Three key findings emerged from this study. First, we did not find significant associations between computational modeling parameters and dimensional parent- and child-rated measures of anxiety and irritability. Second, parent- and child-rated irritability, but not anxiety, tracked with neural responses to face-emotion valence, most notably in the bilateral motor cortex. Finally, we successfully replicated prior behavioral findings of age associations in sensitivity to face-emotion valence and a bias in labeling ambiguous face-emotion in youth with DMDD compared to healthy control youth.

We expected to find an association between clinical dimensional measures of irritability, anxiety, and a bias toward angry judgments of ambiguous face-emotions based on prior work in youth with anxiety disorders and DMDD. Our data do not allow us to draw conclusions as to why we did not find such associations, despite being adequately powered to detect those; but it is of note that some prior work has found diagnostic differences in the absence of associations between dimensionally measured symptoms and bias.^15^ Most youth in our sample met criteria for more than one disorder or reported subthreshold symptoms for another. Thus, in diagnostic analyses, it is difficult to discern which diagnosis/symptom dimension is driving the difference. We did find a significant difference between youth with primary DMDD and healthy controls in perceptual bias in a secondary analysis, pointing cautiously to some specificity. Alternative phenotyping strategies that use continuous approaches, which may also align more closely with underlying neurobiology, may be able to better identify behavioral (and brain–behavior) associations.^31^^,^^49^^,^^50^ Future work should also explore the possibility that perceptual biases associate more globally with measures of clinical impairment, rather than with symptom dimensions. Very few studies can directly speak to causal relations between biases and symptoms,^51^^,^^52^ and interventions targeting biases have found mixed effects.^41^^,^^53^, ^54^, ^55^ Thus, it is plausible that biased social percepts are an epiphenomenon of heightened states of negative arousal and significant functional impairment common to diagnoses of anxiety and irritability.

Localized increased activation to angry faces in the motor cortices associated with higher levels of both parent- and child-rated irritability. This increased neural engagement likely reflects increased vigor in motor responding to overtly angry face-emotions. Additional evidence for this interpretation was derived from supplemental exploratory analyses that showed a correlation between the linear coefficients of neural activation and the computational parameter *t*_0_, indexing non-decision components including motor responding. This increased vigor specifically to angry faces could indicate approach motivation. Heightened physiological arousal in response to perceived threat has been theorized to drive excessive, maladaptive, reactive aggressive responses typical of youth high in irritability. Future work could make use of new tools, such as dynamometers, to directly measure grip strength to quantify approach behavior in response to threat. Interestingly, we did not find associations between either clinical dimension and ambiguity; we expected to see differences in regions implicated in perception and cognitive control. It is possible that measures of connectivity would have been more sensitive to finding more subtle aberrations reflecting awareness or attention to threatening stimulus features. However, with an extremely short presentation of stimuli (150 milliseconds) to specifically target early perceptual processing, our task is suboptimal for timeseries analyses.

We were able to successfully replicate developmental differences in sensitivity along with findings of differences between youth with DMDD and healthy control youth in independent samples, using the same methods as used in the original studies. This increases confidence in the robustness of this association and differences among groups, as well as safeguards against a type I error in the original results. Replication is the foundation of science, yet not necessarily a priority for the scientific community when incentive structures favor original, innovative approaches.

Data attrition and lack of diversity of the sample are significant limitations of this study. Although 95 youth successfully completed the face-emotion labeling task, 160 youth attempted the task. Hence, around 40% of youth were unable to provide data. It was the more severely impaired, younger patients who likely struggled most to sustain attention to complete the task and to tolerate the scanning environment. The face-emotion fMRI task was optimized to increase statistical efficiency and sampling of the hemodynamic response function by using many trials and long, jittered inter-trial intervals; these characteristics also made the task cumbersome to complete. The generalizability of this study’s findings is limited by the sample composition, which consisted of predominately White participants with a household income above the population mean. Finally, our study also used a compound computer-generated face stimulus to create highly controlled representation of face-emotions. When recruiting a more diverse sample of participants, it would be important to use a more diverse stimulus set to explore the impact of stimulus identity on face-emotion labeling, especially given prior findings showing the impact of non-emotional facial features on perceptions of bias.^15^^,^^56^

Co-occurrence of symptoms complicates our understanding of how social cue processing links to important domains of developmental psychopathology. Here we used a computational model to deconstruct perceptual and cognitive components of face-emotion labeling decisions, and fMRI to explore the neural underpinnings of valence and ambiguity face-emotion processing. Future work should consider alternative phenotyping approaches to parse symptoms into meaningful continuous measures to extend this work.

## CRediT authorship contribution statement

**Katherine Y. Kim:** Writing – review & editing, Writing – original draft, Visualization, Validation, Formal analysis, Data curation. **Joel Stoddard:** Writing – review & editing, Resources, Project administration, Methodology, Formal analysis, Data curation, Conceptualization. **Sofia I. Cárdenas:** Writing – review & editing, Project administration, Investigation, Data curation. **Parmis Khosravi:** Writing – review & editing, Software, Formal analysis, Data curation. **Katharina Kircanski:** Writing – review & editing, Project administration, Methodology, Investigation. **Matt Jones:** Writing – review & editing, Visualization, Validation, Software, Formal analysis. **Daniel S. Pine:** Writing – review & editing, Resources, Investigation, Conceptualization. **Melissa A. Brotman:** Writing – review & editing, Supervision, Resources, Funding acquisition. **Simone P. Haller:** Writing – review & editing, Writing – original draft, Visualization, Validation, Supervision, Project administration, Methodology, Investigation, Formal analysis, Data curation, Conceptualization.
