# Supplementary material for: Sensitivity and Bias in Face-Emotion Labeling: Replication and Extension to Youth With Irritability and Anxiety
Source: JAACAP Open. 2025 Oct 22;4(1):42–52. doi: 10.1016/j.jaacop.2025.09.005 (PMC12925860; doi:10.1016/j.jaacop.2025.09.005)
Supplement: Supplementary Material [file mmc1.docx]

Supplementary Materials

**Sensitivity and bias in face-emotion labeling: Replication and extension to youth with irritability and anxiety**

**Demographics for those who contributed to the imaging analyses**

**Table S1. Sample characteristics for the sample of *n*=76**

| **Characteristic** | **Value** |
| --- | --- |
| **Age**, mean (SD) | 14.61 (3.1) |
| **IQ**, mean (SD) | 116.45 (11) |
| **Participant's Household's Gross Income**, No. (%) |  |
| $25,000 - $39,999 | 1 (1.3%) |
| $40,000 - $59,999 | 2 (2.6%) |
| $60,000 - $89,999 | 7 (9.2%) |
| $90,000 - $179,999 | 24 (31.6%) |
| Over $180,000 | 17 (22.4%) |
| Missing | 25 (32.9%) |
| **Highest Level of Education**, No. (%) |  |
| Graduate professional degree- Masters or above | 27 (35.5%) |
| Standard college graduation | 19 (25.0%) |
| Partial college- 1 year or more | 8 (10.5%) |
| High school graduation | 1 (1.3%) |
| Junior high school- 7 to 9 | 2 (2.6%) |
| Less than 7 years of school | 18 (23.7%) |
| Missing |  |
| **Sex**, No. (%) |  |
| Female | 45 (59.2%) |
| Male | 31 (40.8%) |
| **Primary Diagnosis**, No. (%) |  |
| ADHD | 18 (23.7%) |
| Anxiety | 16 (21.1%) |
| DMDD | 20 (26.3%) |
| Healthy | 20 (26.3%) |
| ODD | 2 (2.6%) |
| **Race**, No. (%) |  |
| American Indian or Alaskan Native | 1 (1.3%) |
| Asian | 1 (1.3%) |
| Black or African American | 7 (9.2%) |
| Multiple Races | 7 (9.2%) |
| White | 59 (77.6%) |
| Unknown | 1 (1.3%) |
| **Ethnicity**, No. (%) |  |
| Unknown | 1 (1.3%) |
| Not Hispanic or Latino | 65 (85.5%) |
| Hispanic or Latino | 10 (13.2%) |
| **Medications**, No. (%) |  |
| None | 37 (48.7%) |
| Antidepressants |  |
| SSRI | 17 (22.4%) |
| SNRI | 3 (3.9%) |
| SARI | 1 (1.3%) |
| Mood stabilizers |  |
| AED | 7 (9.2%) |
| Lithium | 1 (1.3%) |
| Anti-anxiety |  |
| Azapirone | 1 (1.3%) |
| Benzodiazepine | 1 (1.3%) |
| SGA | 5 (6.6%) |
| Stimulant | 28 (36.8%) |
| Non-stimulant ADHD | 3 (3.9%) |

*Note*. ADHD = attention deficit/hyperactivity disorder, DMDD = disruptive mood dysregulation disorder, ODD = oppositional defiant disorder.

SSRI = selective serotonin reuptake inhibitory, SNRI = serotonin-norepinephrine reuptake inhibitor, SARI = serotonin antagonist and reuptake inhibitor, AED = antiepileptic drug, SGA = second generation antipsychotic.

*n*=16 participants did not have IQ scores, *n*=25 participants were missing household gross income, *n*=18 participants were missing highest level of education, *n*=1 is missing ethnicity, *n*=7 were missing information about medication use.

Participant’s education and household’s gross income: if the participant is a child or college student, the highest level of education and the income of the parent are considered. If the participant is an adult, their own highest level of education and income are considered.

**SUPPLEMENT 1**

**Addition information on participant assessment and attrition**

The Kiddie-Schedule for Affective Disorders and Schizophrenia for School-Age Children-Present and Lifetime version (KSADS-PL) includes a module for DMDD, which is available on request (Wiggins et al., 2016). Patients presenting with a diagnosis of an anxiety disorder met criteria for generalized anxiety disorder (GAD), separation and/or social phobia and were seeking treatment. Youth with anxiety disorders were additionally excluded for presence of any medication, significant trauma-related symptoms, obsessive compulsive disorder, significant depressive symptoms and suicidal ideation. All diagnoses were confirmed by a senior psychiatrist in a consensus session. Participants were recruited through flyers, mailings, newsletters and approved websites in the community and referrals from local clinics. Travel expenses were reimbursed, and monetary compensation was provided for participation.

160 participants enrolled in the protocol and attempted to complete the task and clinical measures. Of this initial sample, *n*=65 did not provide sufficient good quality data, either because they failed to complete the scan, or they did not meet behavioral thresholds for inclusion. From these 95 participants, 76 participants had imaging data that met motion thresholds.

Data from *n*=24 healthy volunteers were used in a prior publication (Haller et al., 2024).

**Task schematic**

**Figure S1. Task trial schematic**

**Note:** On each trial, a fixation cross preceded the presentation of a face-emotion morph and a mask to remove afterimages and constrain perceptual processing to the short duration of the face-emotion image presentation. Participants indicated via button press whether the face was perceived as predominantly angry or happy. There were 15 possible face-emotion morphs ranging from prototypically happy to angry with middle morphs being the most ambiguous. Face emotion images were presented in random order.

**Quantile probability plots**


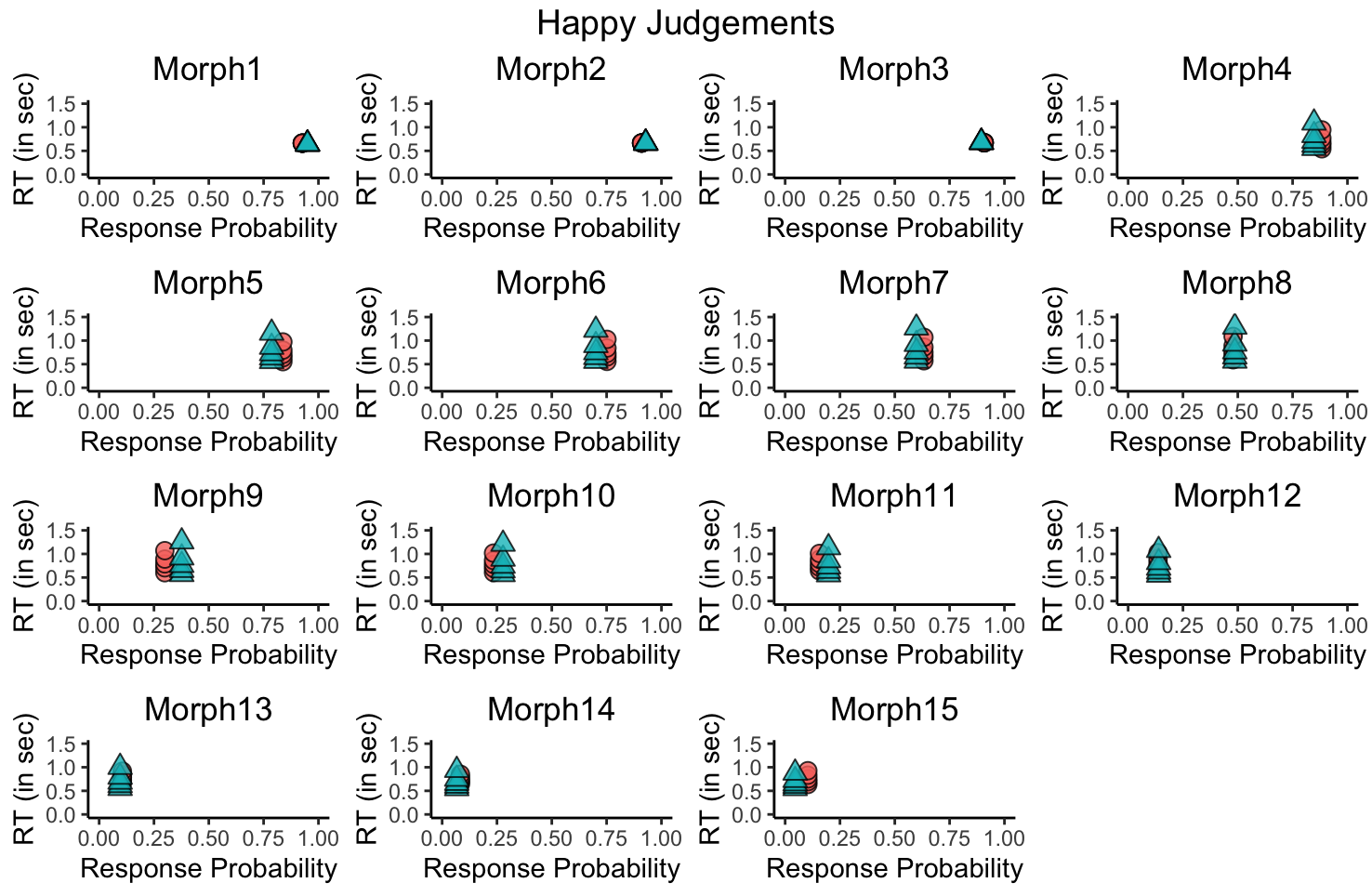

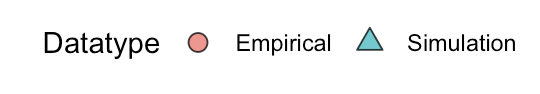

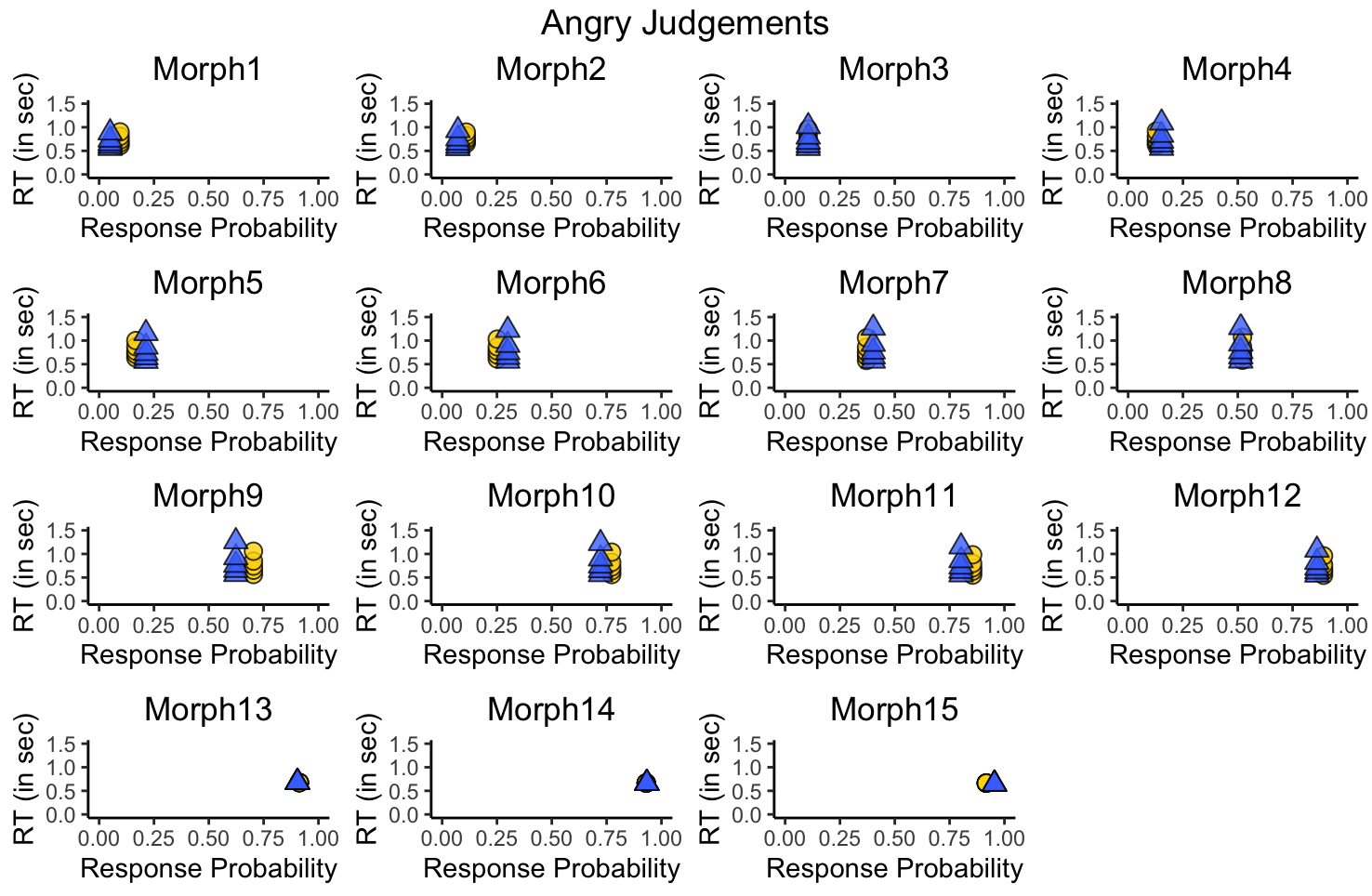

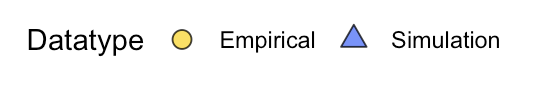


**Figure S2. Quantile probability plots including five reaction time quantiles (0.1, 0.3, 0.5, 0.7, 0.9) for happy (A) and angry (B) judgments for empirical and predicted data**

**Note:** Medians were plotted for errors on the six most extreme morphs as participants made very few inaccurate judgments. The model adequately accounts for accuracy, mean RT and RT variability for the 15 face-emotion morphs.

**Summarizing voxelwise group results after clustering using a “highlighting” approach**

**Table S3. Summary table of significant clusters at a reduced threshold**

| ROI overlap and location | | Cluster Size | | Coordinates (center of mass) | | | Coordinates (at peak) | | | Mean F | SEM | Max Int | Post-hoc^a^ |
| --- | --- | --- | --- | --- | --- | --- | --- | --- | --- | --- | --- | --- | --- |
|  |  | k | mm3 | CM LR | CM PA | CM IS | MI LR | MI PA | MI IS |  |  |  |  |
| *Additional clusters of interest (reduced threshold, p=.01)* | | | |  |  |  |  |  |  |  |  |  |  |
| *Irritability, child-report* | |  |  |  |  |  |  |  |  |  |  |  |  |
| - |  |  |  |  |  |  |  |  |  |  |  |  |  |
| *Irritability, parent-report* | |  |  |  |  |  |  |  |  |  |  |  |  |
| R postcentral gyrus (63.3%), R precentral gyrus (26.6%), R supramarginal gyrus (8.6%) | | 187 | 2921.875 | 52.5 | -16.4 | 45.9 | 42.5 | -14.2 | 65.5 | 10.583 | 0.2724 | 30.538 | 0.315 |
| *Anxiety, child-report* | |  |  |  |  |  |  |  |  |  |  |  |  |
| L precentral gyrus (74.3%), L post central gyrus (25.1%) | | 108 | 1687.5 | -37.8 | -19.5 | 59.1 | -40 | -19.2 | 60.5 | 10.046 | 0.3104 | 20.84 | -0.274 |
| *Anxiety, parent-report* | |  |  |  |  |  |  |  |  |  |  |  |  |
| R middle occipital gyrus (66.0%), R superior occipital gyrus (18.6%), R middle temporal gyrus (9.2%) | | 146 | 2281.25 | 34.7 | -77.6 | 18.1 | 40 | -69.2 | 18 | 9.0894 | 0.1692 | 17.096 | -0.132 |

*Note*. Cluster-corrected voxel-wise linear mixed-effects model results are presented here summarizing regions showing a significant linear trend-by-clinical measure interaction. Location lists regions in descending order based on proportion overlap with cluster. k=number of voxels in cluster, mm3=cluster volume, CM=center of mass of cluster, MI=max intensity (peak), SEM=standard error of the mean, LR=left-right (x), PA=posterior-anterior (y), IS=inferior-superior (z).

^a^Post-hocs represent associations between the individual-level linear coefficient and the clinical measure.

**SUPPLEMENT 2**

**Analyses using reporter-averaged clinical measures**

*Associations between computational parameters and anxiety and irritability, each averaged across reporters*

Anxiety and irritability showed a medium-to-large correlation of *r*_p_(86)=.49, *p*<.001. There were no significant associations between the DDM-derived measures of sensitivity and perceptual bias with either irritability or anxiety (see Table 2 for statistical values) and no other significant associations between estimated parameters (*t*_0_*,z*_r_*,a*) and clinical measures emerged (all |*r*_ps_|<-.07, *p*<.10).

**Table S2. Associations between computational metrics and clinical measures**

|  | Irritability  [*r*_p_(92), *p*-value] | Anxiety  [*r*_p_(86), *p*-value] |
| --- | --- | --- |
| Sensitivity: *v(s)* | -0.065, 0.534 | -0.064, 0.554 |
| Perceptual bias: *s_indiff_* | -0.039, 0.710 | 0.018, 0.869 |

*Associations between neural responses to face-emotion valence and ambiguity and clinical measures*

Only the model examining associations between linear and quadratic activation patterns and irritability revealed significant interactions between the clinical variable and the within-subject slopes at adequate thresholding. Specifically, the left and right primary motor cortex and right cerebellum showed an interaction between irritability and the linear morph term. Post-hoc decomposition of these interactions revealed that neural activation to overt angry morphs increased with increasing levels of irritability [left primary motor: *F*(1,1060)=34.76, *p*<0.001, *r*_p_= 0.368; right primary motor: *F*(1,1060)=25.70, *p*<.001; right cerebellum: *F*(1,1060) =21.720, *p*<0.001].

**
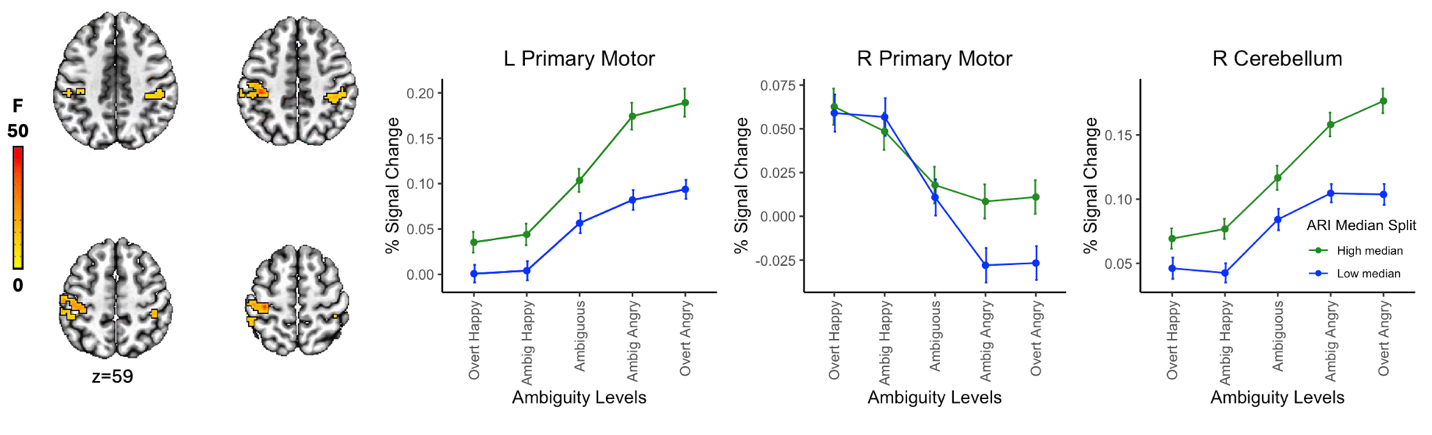
**

**Figure S3.** **Associations between percent signal change to valence (modeled as a linear slope in the voxel-wise model) and irritability in a transdiagnostic sample of youth.**

**Note:** For visualization, a median split was performed on the irritability variable. Signal change estimates are averaged across three adjacent face morphs for display purposes.

**SUPPLEMENT 3**

**Analyses including medication load as an additional covariate**

We repeated the analyses in the main manuscript adding medication load (Sackeim, 2001) as an additional covariate. Seven participants had to be excluded due to missing information on the dosage and duration of medications. Results remained significant with the additional covariate.

| ROI overlap and location | | Cluster Size | | Coordinates (center of mass) | | | Coordinates (at peak) | | | Mean F | SEM | Max Int |
| --- | --- | --- | --- | --- | --- | --- | --- | --- | --- | --- | --- | --- |
|  |  | k | mm3 | CM LR | CM PA | CM IS | MI LR | MI PA | MI IS |  |  |  |
| *Primary clusters (p=.005)* | | |  |  |  |  |  |  |  |  |  |  |
| *Irritability, child-report* | |  |  |  |  |  |  |  |  |  |  |  |
| L postcentral gyrus (65.6%), L inferior parietal lobule (20.3%), L precentral gyrus (10.6%) | | 355 | 5546.88 | -43 | -27.7 | 53.3 | -37.5 | -29.2 | 48 | 15.674 | 0.426 | 51.732 |
| *Irritability, parent-report* | |  |  |  |  |  |  |  |  |  |  |  |
| L postcentral gyrus (72.2%), L precentral gyrus (9.3%), L inferior parietal lobule (7.4%) | | 117 | 1828.13 | -40.2 | -26.2 | 53.5 | -35 | -26.8 | 58 | 12.627 | 0.337 | 28.609 |
| R postcentral gyrus (58.4%), R supramarginal gyrus (21.1%), R inferior parietal lobule (12.2%) | | 95 | 1484.38 | 39.1 | -33.8 | 47.8 | 40 | -34.2 | 50.5 | 10.794 | 0.2734 | 19.194 |
| *Anxiety, child-report* | |  |  |  |  |  |  |  |  |  |  |  |
| - |  |  |  |  |  |  |  |  |  |  |  |  |
| *Anxiety, parent-report* | |  |  |  |  |  |  |  |  |  |  |  |
| - |  |  |  |  |  |  |  |  |  |  |  |  |

**SUPPLEMENT 4**

**Exploratory analyses examining associations between *t*_0_ and neural activation pattern**

Exploratory post-hoc analyses examined associations between the linear coefficients extracted per participant from the three significant clusters derived from the linear-mixed effects model and *t*_0_ using Pearson correlations. *t*_0_ was selected to test the interpretation that significant results in these areas reflected motor execution.

For the model assessing associations with parent-reported irritability, we found a significant association between *t*_0_ and the linear coefficient for the left (*r*_p_(68)=.41, *p*<.001) but not right motor cortex cluster (*r*_p_(68)=.02, *p*>.05). A significant association was also found between *t*_0_ and the linear coefficient for the left motor cortex cluster in the child-reported irritability model (*r*_p_(76)=.33, *p*<.005).

**SUPPLEMENT 5**

**Methods for replications**

Haller et al., 2024: Differentiating neural sensitivity and bias during face-emotion processing in youth: a computational approach

Haller et al., (2024) applied the same computational model to estimate the association between the sensitivity parameter and cross-sectionally assessed age in youth aged 8-38. Following the original publication, we assessed the association between the sensitivity parameter and age using a Pearson correlation.

Stoddard et al., 2016: An Open Pilot Study of Training Hostile Interpretation Bias to Treat Disruptive Mood Dysregulation Disorder

Stoddard et al., (2016) fitted a four-parameter logistic curve (upper and lower limit, slope and inflection point) to group-level (HV, DMDD) happy-angry judgments with face-emotion morph as a continuous variable. The *R* package *drc* was used to fit the curves, which also allowed for a testing of differences between parameters. Only differences in the inflection point were examined, termed “balance point” in the prior publication, representing the point on the curve where judgments switched from predominantly happy to angry, adjusted for upper and lower limits (i.e., maximum probability of either judgment). The lower the inflection point, the more of an “angry bias” or “hostile interpretation bias” the participant exhibits. All procedures were kept identical to the original publication.

**Additional references:**

Haller, S. P., Stoddard, J., Cardenas, S. I., Dombek, K., MacGillivray, C., Botz-Zapp, C., Bui, H. N. T., Stavish, C. M., Kircanski, K., & Jones, M. (2024). Differentiating neural sensitivity and bias during face-emotion processing in youth: a computational approach. *Social Cognitive and Affective Neuroscience*, *19*(1), nsae034.

Sackeim, H. A. (2001). The definition and meaning of treatment-resistant depression. *Journal of Clinical Psychiatry*, *62*, 10-17.
